# Runx2 mediates epigenetic silencing of the bone morphogenetic protein-3B (BMP-3B/GDF10) in lung cancer cells

**DOI:** 10.1186/1476-4598-11-27

**Published:** 2012-06-18

**Authors:** Manish Tandon, Karthiga Gokul, Syed A Ali, Zujian Chen, Jane Lian, Gary S Stein, Jitesh Pratap

**Affiliations:** 1 Department of Anatomy and Cell Biology, Rush University Medical Center, Armour Academic Center, 600 S. Paulina St. Suite 507, Chicago, IL, 60612, USA; 2 Departments of Cell Biology, and Cancer Center, University of Massachusetts Medical School, Worcester, MA, USA

**Keywords:** Lung cancer, Runx2, BMP-3B, Gene silencing

## Abstract

**Background:**

The Runt-related transcription factor Runx2 is essential for bone development but is also implicated in progression of several cancers of breast, prostate and bone, where it activates cancer-related genes and promotes invasive properties. The transforming growth factor β (TGF-β) family member bone morphogenetic protein-3B (BMP-3B/GDF10) is regarded as a tumor growth inhibitor and a gene silenced in lung cancers; however the regulatory mechanisms leading to its silencing have not been identified.

**Results:**

Here we show that Runx2 is highly expressed in lung cancer cells and downregulates BMP-3B. This inverse relationship between Runx2 and BMP-3B expression is further supported by increased expression of BMP-3B in mesenchymal cells from Runx2 deficient mice. The ectopic expression of Runx2, but not DNA binding mutant Runx2, in normal lung fibroblast cells and lung cancer cells resulted in suppression of BMP-3B levels. The chromatin immunoprecipitation studies identified that the mechanism of Runx2-mediated suppression of BMP-3B is due to the recruitment of Runx2 and histone H3K9-specific methyltransferase Suv39h1 to BMP-3B proximal promoter and a concomitant increase in histone methylation (H3K9) status. The knockdown of Runx2 in H1299 cells resulted in decreased histone H3K9 methylation on BMP-3B promoter and increased BMP-3B expression levels. Furthermore, co-immunoprecipitation studies showed a direct interaction of Runx2 and Suv39h1 proteins. Phenotypically, Runx2 overexpression in H1299 cells increased wound healing response to TGFβ treatment.

**Conclusions:**

Our studies identified BMP-3B as a new Runx2 target gene and revealed a novel function of Runx2 in silencing of BMP-3B in lung cancers. Our results suggest that Runx2 is a potential therapeutic target to block tumor suppressor gene silencing in lung cancer cells.

## Background

Lung cancer is the leading cause of cancer mortality and accounts for 30% of all deaths from cancer [1]. Silencing of tumor suppressor genes by aberrant promoter hypermethylation is a key event in lung cancer initiation and progression. During gene silencing, the chromatin structure is altered by acetylation, phosphorylation and methylation of histone tails [2]. These alterations in chromatin structure affect normal cell functions and are a crucial trigger for neoplastic development and progression [3]. However, current understanding of regulatory mechanisms of silencing of tumor suppressors is limited. In this study we identified a mechanism by which Runx2 transcription factor contribute to epigenetic silencing of a tumor growth inhibitor BMP-3B in lung cancer cells.

Runx transcription factors (Runx1, Runx2 and Runx3) are critical regulators of organogenesis and cell differentiation regulatory pathways, and mutations in these genes are associated with several cancers. Runx2, an essential bone cell differentiation factor [4,5] is recently implicated in mammary, prostate and osteosarcoma progression [6-8]. In cancer cells, Runx2 activates cancer-related genes, promotes cells invasive properties [6,8-10], cooperates with oncogenes (e.g., c-myc in T-cell lymphoma development), and suppresses apoptotic and growth arrest pathways [11,12]. Runx2 is also a major target gene of TGFβ /BMP signaling pathway and the interaction between Runx2 and Smads results in regulation of downstream target genes in osteoblasts [13], chondrocytes [14] and cancer cells [8].

BMP-3B, a TGFβ family member and closely related to BMP-3, is highly expressed in lung [15-17], brain and bone tissues, and induces bone formation [18,19]. Ectopic BMP-3B expression promotes osteoblast differentiation and augments the bone formation induced by bone morphogenetic protein-2 (BMP-2) in rats [20]. Importantly, the expression of BMP-3B is downregulated in lung cancer patient samples and cancer cells lines compared to normal lung cells [21-23]. Multiple mechanisms have been proposed for the downregulation of BMP-3B levels which include methylation of gene promoter and repression by transcription factors [21] however, the transcriptional repressor proteins of BMP-3B are unknown.

We show that BMP-3B is a novel Runx2 target gene and find an inverse relationship between Runx2 and BMP-3B expression levels in normal lung fibroblast and lung cancer cells. Our studies with Runx2 overexpression or knockdown in lung cancer cells indicate that Runx2-mediated downregulation of BMP-3B is via increasing histone H3K9 methylation status of the proximal promoter by interacting with methyltransrefase Suv39h1.

## Results

### Calvarial mesenchymal cells of Runx2-deficient mice have higher expression levels of BMP-3B

**Figure 1:**
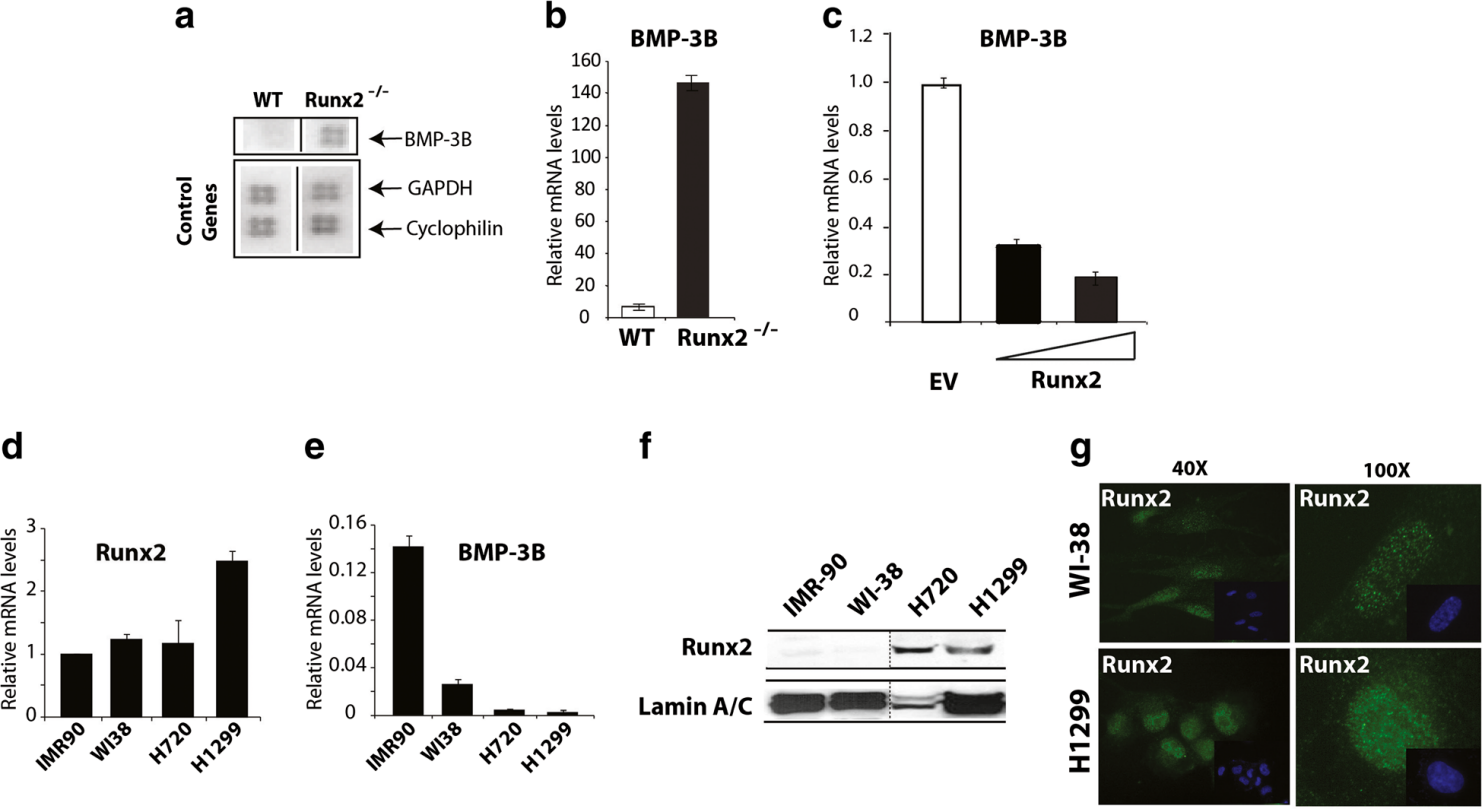
**Runx2 deficient mice have higher levels of BMP-3B. (a)** Total RNA from mesenchymal cells of calvarial tissue from wild type and Runx2^-/-^ (17.5 dpc) was hybridized with osteogenesis-related cDNA array. Signal of BMP-3B on blots is indicated by an arrow. Control genes (GAPDH, cyclophilin A) are shown in lower panel. (**b**) BMP-3B mRNA levels (normalized to GAPDH) in calvarial mesenchymal cells from wild type (WT) and Runx2^-/-^ animals as detected by qRT-PCR analysis. (**c**) Primary calvarial cells from Runx2 deficient animals were transduced with empty vector (EV) or Runx2 expressing adenovirus with increasing multiplicity of infection (MOI 10 and 20) for 48 hr. Total RNA isolated from the infected cells was utilized to examine BMP-3B mRNA as detected by qRT-PCR analysis. (**d**) Runx2 mRNA expression levels were examined by qRT-PCR and the gene expression levels were normalized to 28S internal control. The gene expression levels were calibrated to IMR-90 cells. (**e**) BMP-3B mRNA expression levels were examined by qRT-PCR and the gene expression levels were normalized to 28S internal control. The BMP-3B gene expression levels were calibrated to Runx2 levels in IMR-90 cells. (**f**) Runx2 protein levels were examined in nuclear extracts of normal lung fibroblast cells (IMR-90 and WI-38) and lung cancer cell lines (H720 and H1299) by immunoblotting with Runx2 monoclonal antibody. Runx2 expression levels were normalized to internal control LaminA/C protein. (**g**) Runx2 intracellular localization was determined by immunofluorescence of endogenous Runx2 protein in WI-38 and H1299 cells. The punctate (Alexa 488, green) signal shows Runx2 staining while nuclei are revealed by 4, 6-diamidino-2-phenylindole (DAPI, blue) staining.

To identify novel Runx2 target genes, we performed cDNA expression analysis on total RNA isolated from calvarial mesenchymal cells of wild type and functional deficient Runx2 mice [5]. In addition to the downregulation of known Runx2 target genes (e.g., matrix metalloproteinases) in a osteogenesis-related cDNA array [24], we found that the expression levels of BMP-3B gene was induced in Runx2 deficient cells compared to wild type cells (Figure 1a). The induction of BMP-3B expression in Runx2 deficient calvarial mesenchymal cells was validated by qRT-PCR analysis (Figure 1b). To further confirm Runx2-mediated downregulation of BMP-3B levels, we re-expressed Runx2 via adenoviral delivery in Runx2 deficient primary calvarial cells and measured BMP-3B levels by qRT-PCR analysis (Figure 1c). Our results show a dose-dependent repression of BMP-3B mRNA levels by Runx2 in primary osteoblastic cells. These results suggested that BMP-3B is a novel Runx2 responsive gene.

### An inverse relationship between Runx2 and BMP-3B expression levels in lung cancer cells

A tumor growth inhibitory function was proposed for BMP-3B in lung cancers and BMP-3B is downregulated in most of the lung cancers [22,23,25]. In context of Runx2-mediated BMP-3B suppression in mesenchymal cells and to understand the upstream regulatory mechanisms of BMP-3B silencing in lung cancers, we hypothesized that Runx2 downregulates BMP-3B expression in lung cancer. To understand the role of Runx2 in BMP-3B transcriptional regulation in lung cancer cells, we first examined Runx2 and BMP-3B mRNA levels in normal lung fibroblasts of mesenchymal origin (WI-38 and IMR-90), atypical carcinoid (H720) and metastatic non-small cell lung carcinoma (H1299) cells by qRT-PCR analysis. Our results showed that Runx2 expression is increased in metastatic lung cancer cells (H1299) compared to normal lung fibroblast cells. In contrast to the Runx2 expression levels, BMP-3B mRNA was detectable but lower in lung cancer cells compared to normal lung fibroblast cells (Figure 1d and e). The Western blot analysis for Runx2 protein levels further validated increased Runx2 levels in lung cancer cells compared to normal lung fibroblast cells (Figure 1f). A punctate nuclear staining of Runx2 was observed in WI-38 and H1299 cells as examined by immunofluorescence (Figure 1g). Taken together, these studies revealed that the inverse relationship between Runx2 and BMP-3B levels observed in calvarial mesenchymal cells also holds true for normal lung fibroblasts and lung cancer cells.

### Runx2 overexpression suppresses BMP-3B in lung cancer cells

**Figure 2:**
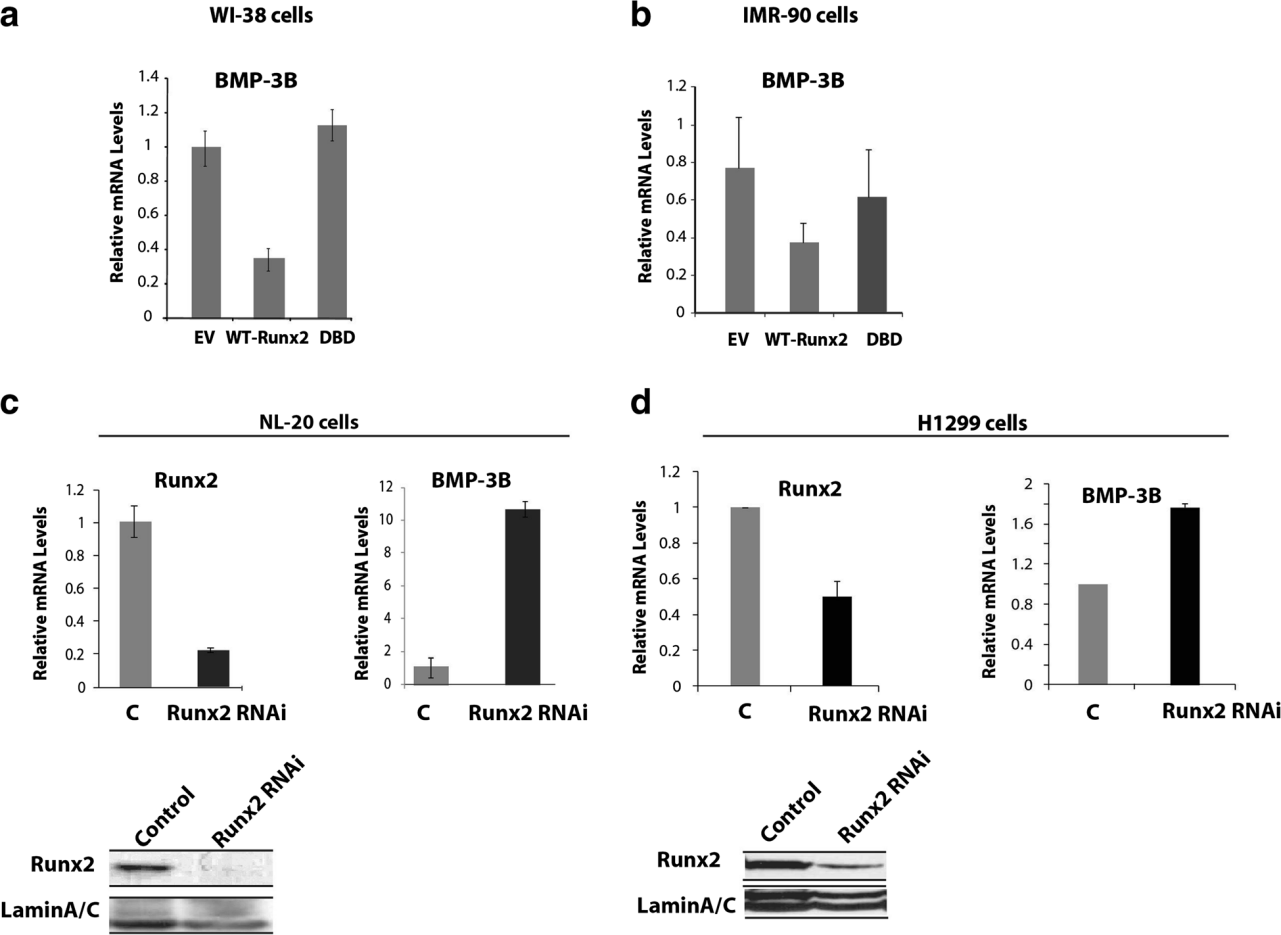
**Runx2 suppresses BMP-3B in lung cancer cells. (a and b)** Expression levels of BMP-3B in normal lung fibroblast cells (**a**: WI-38, **b**: IMR-90) transduced with wild type Runx2 (WT), functional deficient mutant (DBD: DNA binding domain mutant,) and empty vector (EV) control lentiviral particles are shown as detected by qRT-PCR analysis. (**c and d**) The Runx2 protein was suppressed in normal bronchial NL-20 epithelial cells and H1299 lung cancer cells by lentiviral vector-mediated RNAi. The suppression of Runx2 at mRNA and protein expression levels is shown for NL-20 cells (C top left and lower left panels). The mRNA levels of BMP-3B in Runx2 suppressed NL-20 and control cells are shown as examined by qRT-PCR (C top right panel). The suppression of Runx2 at mRNA and protein expression levels is shown for H1299 cells (D top left and lower left panels). The mRNA levels of BMP-3B in Runx2 suppressed H1299 and control cells as examined by qRT-PCR (D top right panel).

To investigate whether Runx2 suppresses BMP-3B levels in lung cancer cells similar to observed in primary calvarial cells, we stably overexpressed wild type Runx2 (WT) and Runx2 DNA binding domain mutant (DBD) in normal lung fibroblast cells (WI-38 and IMR-90) by lentiviral-mediated gene delivery. Expression levels of wild type and mutant Runx2 protein in these cell types were confirmed by qRT-PCR and western blot analysis (data not shown). Our results showed that stable expression of wild type Runx2 in normal lung cells resulted in more than 2-fold decrease in BMP-3B levels compared to empty vector control cells (Figure 2a and b). Ectopic expression of DBD mutant of Runx2 failed to downregulate BMP-3B levels in normal lung or lung cancer cells. These results suggested that the Runx2 DNA binding activity is required for BMP-3B regulation. In complementary studies, Runx2 knockdown resulted in increased BMP-3B levels in normal bronchial NL-20 cells (10- fold increase, Figure 2c right panel) and H1299 cells (2-fold increase, Figure 2d right panel) compared to empty vector controls as shown by qRT-PCR analysis. The decrease in Runx2 levels in Runx2 knockdown cells was confirmed by qRT-PCR and western blot analysis (Figure 2c and d). Collectively, these results indicate that Runx2 downregulates BMP-3B levels in normal lung fibroblast and lung cancer cells.

### Runx2 recruitment on the BMP-3B gene promoter and interaction with Suv39h1 promotes BMP-3B silencing

**Figure 3:**
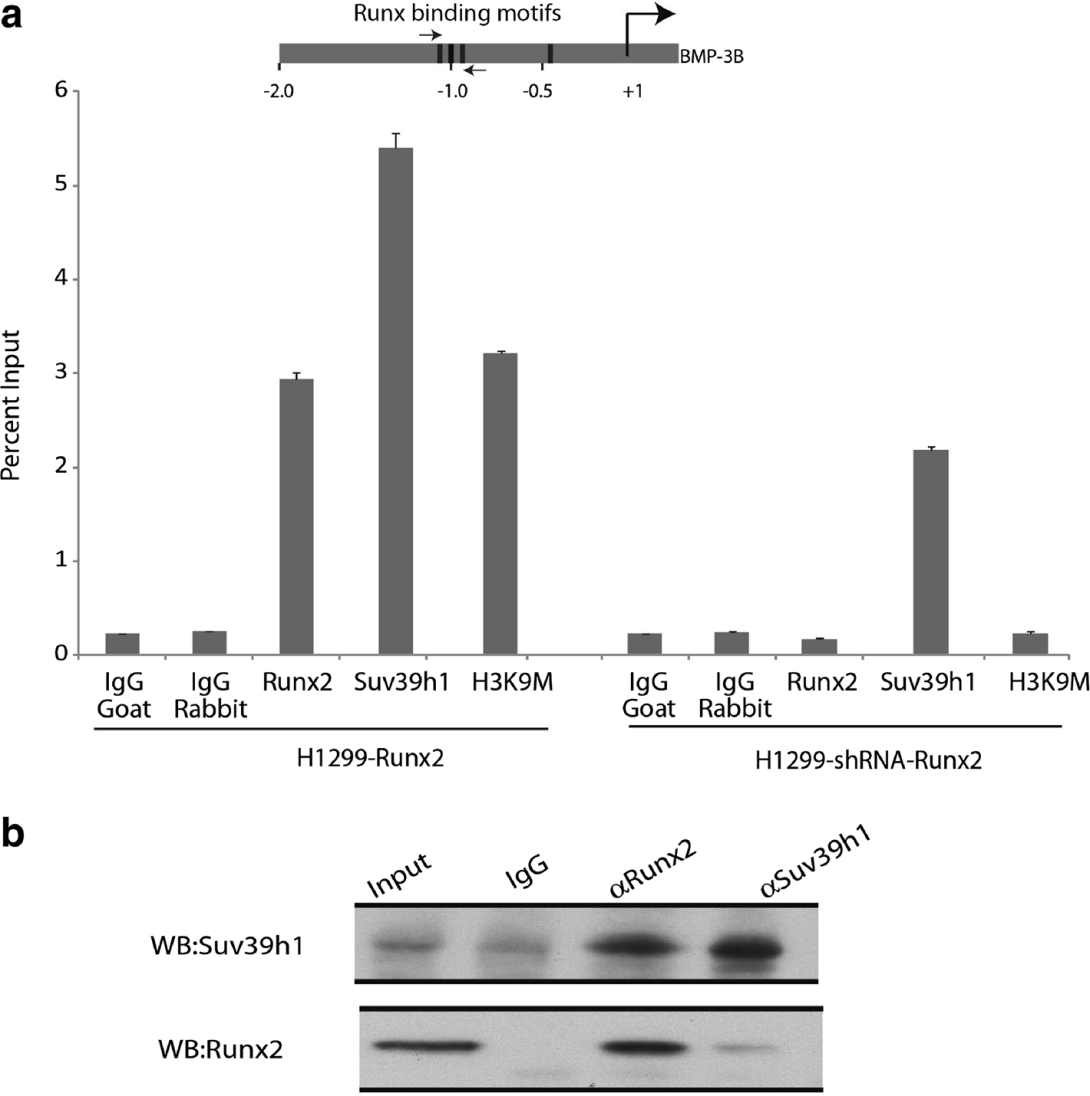
**Runx2 recruitment on BMP-3B gene promoter and interaction with Suv39h1.** (**a**) Lung cancer cells (H-1299) stably expressing wild type Runx2 (left panel) or shRNA-Runx2 (right panel) were subjected to chromatin immunoprecipitation assay with Runx2, Suv39h1, H3K9 or control IgG-G (goat) and IgG-R (rabbit) antibodies. Immunoprecipitated DNA samples were further amplified by real-time qPCR with primer pairs for proximal promoter (-1.0kb) of BMP-3B gene. A schematic diagram above indicates Runx binding motifs (solid bars) on BMP-3B promoter. The BMP-3B ChIP primer pairs are indicated by arrows in the schematic diagram. (**b**) Co-immunoprecipitation assay shows Runx2 interaction with Suv39h1 in H-1299 lung cancer cells. Whole cell extracts from lung cancer H-1299 cells were incubated with Runx2, Suv39h1 or control IgG antibodies. Western blots showing Runx2 and Suv39h1 proteins as detected by αRunx2 and αSuv39h1 antibodies.

To further investigate the mechanism of Runx2-mediated downregulation of the BMP-3B expression in lung cancer cells, we performed chromatin immunoprecipitation analysis in H1299 cells expressing either wild type Runx2 or shRunx2 (Figure 3). Our results showed 3-fold increased Runx2 binding on the BMP-3B proximal promoter (-1.0kb) in H1299-WT-Runx2 cells, that was abrogated in H1299-shRunx2 cells. We next examined the methylation status of the BMP-3B proximal promoter as methylation of lysine 9 of histone H3 (H3K9) allows the binding of heterochromatin protein- 1 (HP1) to silence gene expression [26,27]. Our results show increased (3-fold) H3K9 levels of proximal promoter region of BMP-3B in H1299-Runx2 cells compared to H1299-shRunx2 cells or antibody controls (Figure 3a). We next examined the recruitment of Suv39h1 protein, a histone H3 lysine 9 specific methyltransferase, on BMP-3B proximal promoter. A twofold increase in recruitment of Suv39h1 was observed in H1299-Runx2 cells compared to H1299-shRunx2 lung cancer cells (Figure 3a). These findings indicated the possibility of physical interaction of Runx2 and Suv39h1 proteins in lung cancer cells. We performed co-immunoprecipitaion assays with Runx2 and Suv39h1 antibodies and a direct interaction of Runx2 with Suv39h1 proteins was detected in H1229 cells (Figure 3b). Taken together, these results show that the recruitment of Runx2 and Suv39h1 on the BMP-3B proximal promoter sequences resulted in increased H3K9 methylation status and consequently downregulation of BMP-3B expression in lung cancer cells.

### Runx2 increases wound-healing response of lung cancer cells

**Figure 4:**
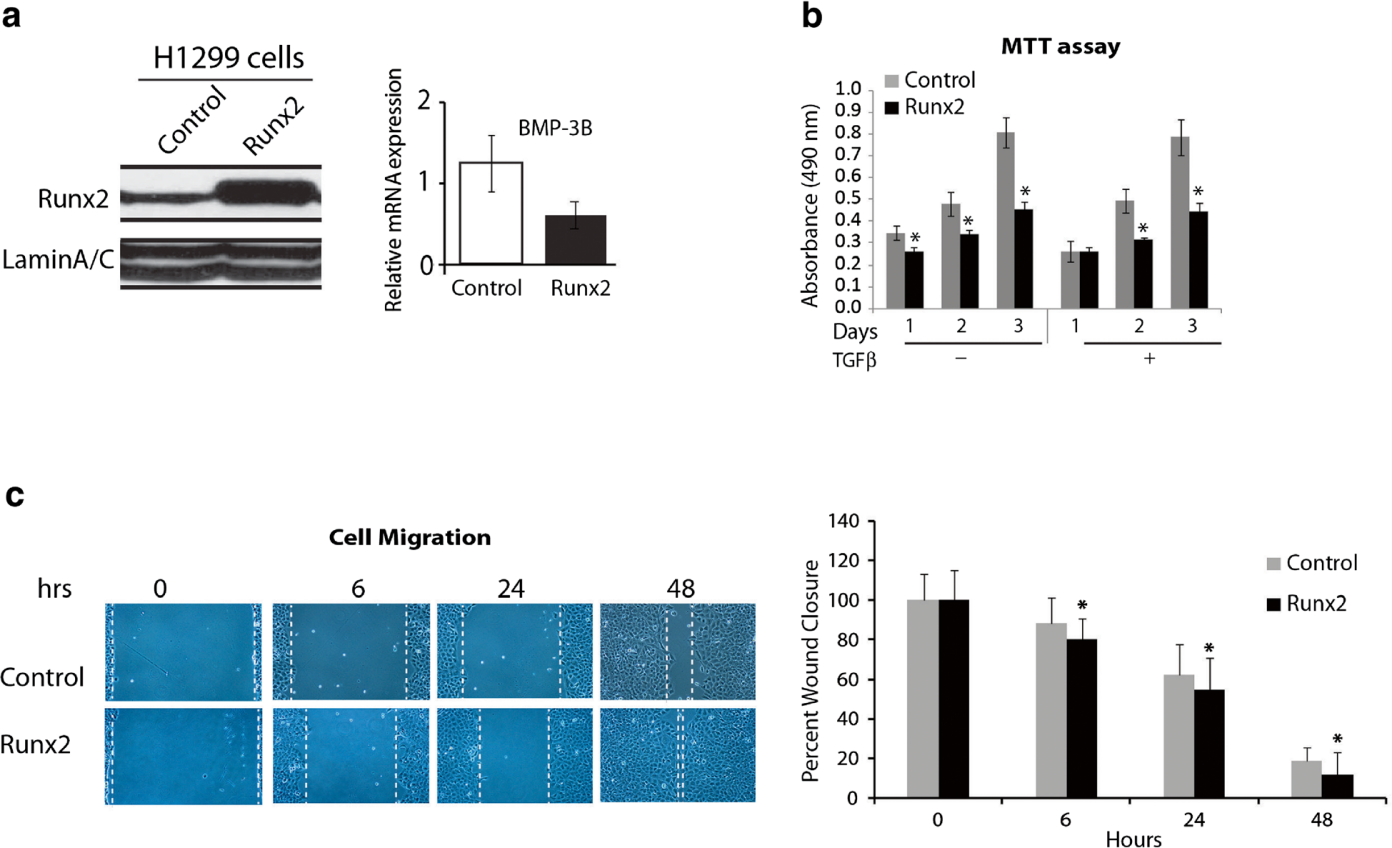
**Runx2 increases migration potential of lung cancer cells. (a)** Left panel: Runx2 protein was overexpressed in H-1299 cells via lentiviral vector-mediated gene transfer and overexpression levels are shown by Western blot analysis. Right panel**:** The mRNA levels of BMP-3B in Runx2 overexpressed H1299 cells as examined by qRT-PCR **(b)** The Runx2 overexpressing H1299 cells or empty vector (control) were seeded in a 96 well cell culture dish in presence or absence of TGFβ (5ng/ml) and examined for changes in cell proliferation via cell proliferation MTT assay by measuring absorbance at 490 nm. **(c)** Cell migration in H-1299 cells expressing Runx2 or empty vector control was examined in a standard wound/scratch healing assay in response to TGF-β treatment. Representative images at 40X magnification and average (±standard deviation) wound healing response is shown at indicated time points. (* P ≤ 0.05).

To examine the phenotypic effects of Runx2 overexpression in lung cancer cells, we assessed proliferation and migration potential of H1299-Runx2 cells or H1299- empty vector cells. Increased Runx2 levels in H1299-Runx2 cells and a corresponding decrease in BMP-3B mRNA expression were confirmed by western blot and qRT-PCR analysis respectively (Figure 4a). A 40% decline in cell proliferation was observed in Runx2 overexpressing H1299 cells compared to empty vector control cells in absence or presence of TGFβ treatment as examined by cell growth assay (data not shown) and MTT assays (Figure 4b). However, in response to TGF-β treatment the Runx2 overexpression in H1299 cells resulted in a significant (p <0.05) increase in wound healing response compared to the empty vector control for 6-48h as shown by wound healing assay (Figure 4c). The H1299 EV or WT-Runx2 cells did not show any differences in KI-67 (cell proliferation marker) immunoreactivity around wound area (data not shown). These results suggest that Runx2 promotes migratory potential of lung cancer cells by modulating TGF-β/BMP-3B signaling axis.

## Discussion

Our studies identify BMP-3B as a Runx2 target gene and show that Runx2 promotes epigenetic silencing of BMP-3B in lung cancer cells by promoting histone H3K9 methylation status of the proximal regulatory regions. The Runx2 interaction with Suv39h1 methyltransferase and binding to the BMP-3B promoter results in downregulation of the BMP-3B expression levels. Furthermore, ectopic expression of Runx2 enhances the migration potential of lung cancer cells in response to the TGFβ signaling.

We find that mesenchymal cells from Runx2-deficient animals express high levels of BMP-3B compared to wild type cells. In contrast to high levels of BMP-3B, low baseline levels of BMP2 are reported in Runx2 deficient cells that can be up-regulated by ectopic expression of Runx2 [28]. Interestingly, a BMP2 orthologous signaling antagonizing function for BMP3/3B has been proposed during embryonic development of xenopus [29]. In addition to directly regulating expression levels of BMP family members as shown by these studies, Runx2-Smad complex has been shown to regulate expression of genes related to osteogenic [10,30,31] and cancer [32] properties in response to TGFβ/BMP signaling. The consequences of direct regulation of BMP-3B by Runx2 on downstream molecular events of TGFβ/BMP pathway still need to be determined. A recent report shows that the migration of lung cancer cells is associated with the upregulation of Runx2 and Snail expression in response to BMP-2 treatment [33]. Our results show that Runx2 downregulates BMP-3B and increases migration potential of lung cancer cells in response to TGFβ treatment. These studies suggest that cross-talk between Runx2 and TGFβ/BMP signaling is differential and could be context-dependent.

Our results showing higher gene and protein expression levels of Runx2 in lung cancer cells compared to normal lung fibroblast cells are consistent with previous reports of Runx2 expression in other epithelial cancers like breast and prostate cancers [8,10,31,34-36]. The Runx2 gene expression levels were similar in IMR-90 and WI-38 cells; however BMP-3B levels were dramatically reduced suggesting cell-type-specific differences. In addition, we find that the Runx2 overexpression in lung cancer cells results in a significant decline in cell proliferation but enhances wound healing response. In serum-deprived conditions used for the wound healing assay, we observed similar numbers of KI-67 positive cells close to (200-400μm) wound area in both EV and WT-Runx2 over-expressing cells. As we find KI-67 positive cells in both groups, therefore, we cannot completely rule out the possible contribution of cell proliferation in the observed wound healing phenotype. This phenotype is probably the combinatorial effect of Runx2 on BMP-3B suppression and activation of genes related to invasion and migration (e.g., MMPs), as Runx2 is known to promote migration and invasive potential of breast and prostate cancer cells [6,8,9,24,33,35,37-39]. The downstream molecular events of BMP-3B silencing in lung cancer progression are still not clear and might include phosphorylation of Smad proteins as recently reported that BMP-3B inhibits tumor formation of mammary tumor cells by promoting phosphorylation of Smad3[40].

An important finding of our study is the identification of mechanism where Runx2 protein downregulates BMP-3B levels by interacting and recruitment of Suv39h1 methyltransferase at the proximal regulatory sequence. Similar to our findings, a direct interaction of Suv39h1with the C-terminal domain of other Runx family members (Runx1 and Runx3) results in silencing of CD4 gene by promoter methylation during T-cell development [41,42]. Runx2 is well known to regulate chromatin structure and modulate target gene expression [43]. For example, Runx2 interaction with p300 alters chromatin structure (acetylation of histones H3 and H4) during activation of MMP-13 gene in bone cell lineage in response to PTH [44] and enhances histone acetylation resulting in increased Snail expression and decreased E-cadherin in lung cancer cells [33]. Recent reports indicate that Runx2 forms complexes containing the RNA Pol I transcription factors UBF1 and SL1, co-occupies the rRNA gene promoter with these factors in vivo, and affects local chromatin histone modifications at rDNA regulatory regions during rDNA suppression [45,46]. Consistent with these studies, our results revealed that Runx2 regulates histone H3K9 methylation status of BMP-3B promoter in lung cancer cells. There is a possibility that Runx2 repressor complex on BMP-3B promoter includes members of HDAC family as previously shown for repressing bone sialoprotein gene expression in osteoblastic lineage cells [47-49].

**Figure 5:**
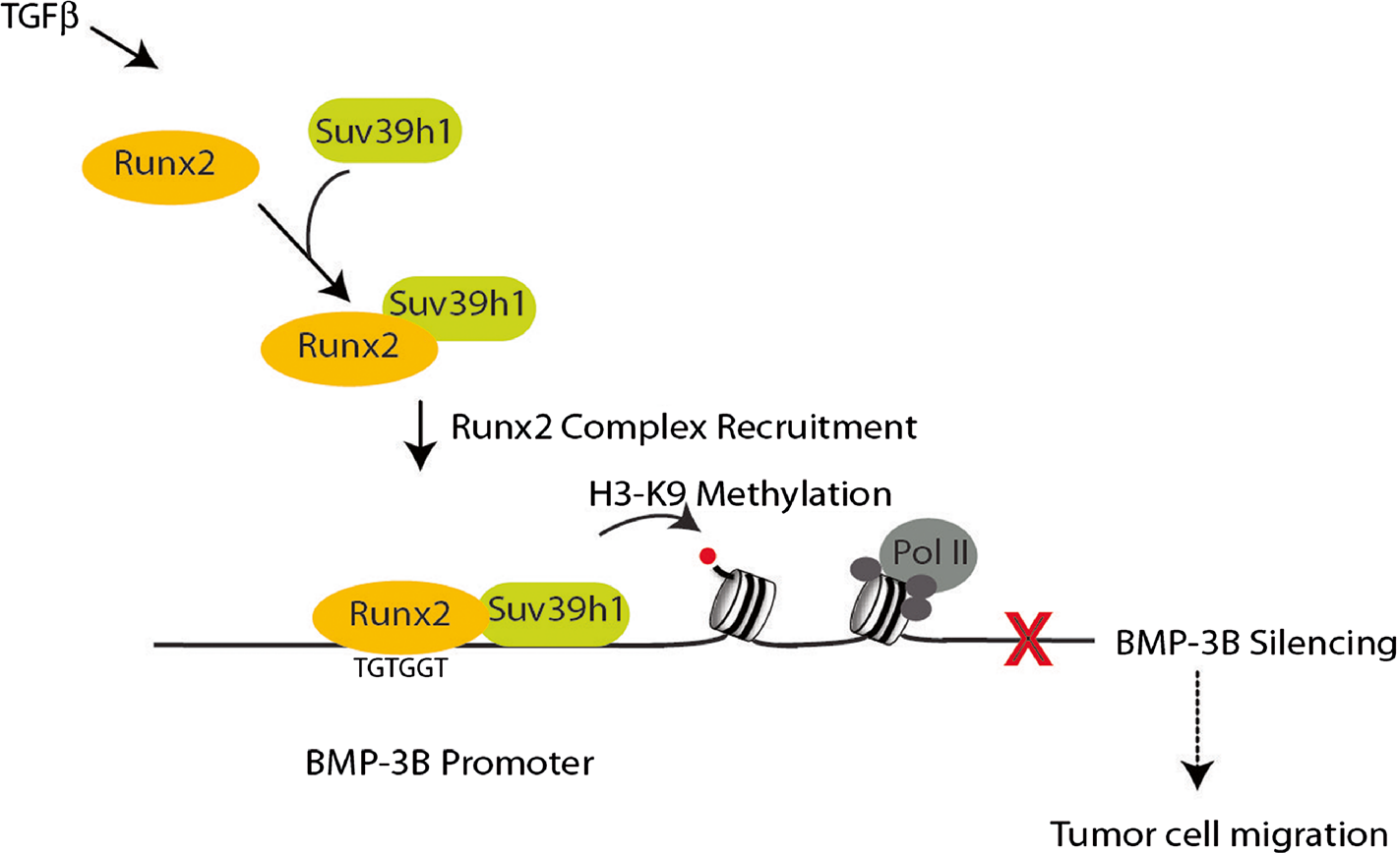
**Illustration of Runx2-mediated epigenetic silencing of BMP-3B in lung cancer cells.** The recruitment of Runx2 and Suv39h1 on the BMP-3B locus increases histone H3K9 methylation status resulting in downregulation of BMP-3B expression in lung cancer cells.

In summary, our study demonstrates BMP-3B as a novel target gene for Runx2 in bone lineage and lung cancer cells and provides insight into mechanisms that regulate epigenetic silencing of tumor growth inhibitors in lung cancer cells (Figure 5). Further studies are required to definitely establish the contribution of Runx2 in lung cancer progression.

## Conclusions

Taken together, our results identified BMP-3B as a new Runx2 target gene and revealed a novel function of Runx2 in epigenetic silencing of BMP-3B in lung cancer cells. Our studies with modulation of Runx2 levels in lung cancer cells indicate that Runx2-mediated downregulation of BMP-3B levels is via interacting with methyltransrefase Suv39h1 and increasing histone H3K9 methylation status of the proximal promoter. These results suggest that Runx2 is a potential therapeutic target to block tumor suppressors gene silencing in lung cancer cells.

## Materials and methods

### Cell Culture and treatments

Normal bronchial and lung fibroblast (NL-20; WI-38, and IMR-90) and lung cancer cells (small cell lung cancer and non-small cell lung cancer cells: H720, and H1299) were cultured in growth medium as specified by American Type Culture Collection. The construction and procedure for wild type Runx2 or DNA binding mutant expressing adenovirus and lentivral transduction in normal and cancer cells are reported previously [24,45].

### Animal procedures

Animals were maintained at the University of Massachusetts Medical School following procedures approved by the Institutional Animal Care and Use Committee (IACUC). Primary calvarial cells from Runx2 ^-/-^ mice were isolated as previously described [24].

### shRNA treatment

Normal bronchial NL-20 or lung cancer H-1299 cells were transduced with lentivirus expressing shRNA-Runx2 target sequence 5’-AAGGTTCAACGATCTGAGATTTG-3’ sequence in pLVTHM vector under H1 promoter [34]. Runx2 knockdown efficiency was confirmed by western blot and real time RT-PCR analysis.

### Western blot analysis

Runx2 protein levels in normal bronchial, fibroblast and lung cancer whole cell lysates or nuclear lysates were detected by western blot analysis as described previously [24]. Runx2 antibody (MBL Inc., Woburn, MA) or Suv39h1 (C-14, Santa Cruz Biotechnology Inc. Santa Cruz, CA) and HRP-conjugated secondary antibodies (Santa Cruz) were used to detect immunoreactive proteins.

### Chromatin immunoprecipitation

Chromatin immunoprecipitation (ChIP) was performed as previously described [34]. Protein-DNA complexes were immunoprecipitated using Runx2 antibody (M-70, Santa Cruz Biotechnology Inc.), Suv39h1 (Santa Cruz Biotechnology Inc.) and histone H3K9 (Abcam, Cambridge, MA) or IgG as a control. Purified DNA was subjected to real time PCR amplification with SYBR Green chemistry on an ABI real time thermocycler. BMP-3B promoter fragment containing Runx elements were amplified using forward primer: 5’ ACT TTG ATG AAT CCG CAA CC-3’ and reverse primer: 5’ TTG TCT TGC CTC TAGCAG GAT-3’.

### Real time RT-PCR analysis

The mRNA levels of Runx2, BMP-3B, GAPDH and 28S in primary osteoblasts, normal lung fibroblast, bronchial and lung cancer cells were analyzed after adenovirus- or lentiviral-mediated Runx2 transduction. Total RNA was isolated using Trizol reagent (Invitrogen, Carlsbad, CA) according to the manufacturer’s specification. Purified RNA was oligo dT primed and cDNA synthesized at 42°C with SuperScript II RNA polymerase (Invitrogen). For PCR amplification, the following primers were used: Runx2, forward primer: 5’- CGG CCC TCC CTG AAC TCT -3’, reverse primer: 5’- TGC CTG CCT GGG GTC TGT A -3’, GAPDH, forward primer: 5’- ATG TTC GTC ATG GGT GTG AA -3’, reverse primer: 5’- TGT GGT CAT GAG TCC TTC CA -3’. BMP-3B, forward primer: 5’-AGC TGC TGG ACT TTG ACG AG-3’, reverse primer: 5’-TGA CAA TGC TCT GGA TGG TG-3’. 28S, forward primer: 5’- GAA CTT TGA AGG CCG AAG TG-3’, reverse primer: 5’-ATC TGA ACC CGA CTC CCT TT-3’. The gene expression levels were quantified by ΔΔCt method of relative quantification by normalizing the data with internal control and expressed relative to appropriate control cell line as indicated in the figure legends.

### Wound healing assay

H1299 cells stably expressing Runx2 or empty vector treated control cells were cultured in triplicates in a 6 well dish with reduced serum conditions (0.2% serum) for overnight. The next day, a scratch was made approximately in the center of the monolayer by a sterile 200μl pipette tip. The detached cells and debris were washed with serum-free RPMI medium. The cells were then supplemented with or without TGF-β (5 ng/ml) containing RPMI medium. Five random images per well were photographed at 0h, 6h, 24h and 48h. The distance of the scratch was measured in ImageJ software at every time point. The wound distance at 0h was assigned as 100% and used to calculate percent wound closure at other time points. The P-value for statistical significance was calculated by unpaired T-test.

### Cell proliferation assay

H1299 cells stably expressing Runx2 or empty vector treated control were counted in a hemacytometer and 1000 cells per well were seeded in a 96-well plate. To determine the changes in proliferation, the cells were indirectly assayed for cell number via a tetrazolium compound-based colorimetric assay (CellTiter 96 kit from Promega Inc. Madison, WI) according to manufacturer’s instructions. At indicated time points over a period of four days, the cell titer reagent (20μl/well) was added to the plate and incubated at 37°C for 1 hour. The quantity of color developed (formazan product from the tetrazolium compound) was measured by reading absorbance at 490 nm in a spectrophotometer (Fluostar Optima BMG Labtech Inc. Cary, NC).

### Immunoprecipitation

Lung cancer H1299-WT-Runx2 or -shRunx2 cells were washed with ice-cold PBS and harvested in lysis buffer [50 mM NaCl, 50 mM Tris (pH 8.0), 1% NP- 40, 25 mM MG132, and 1× protease inhibitor mixture (Roche, Indianapolis, IN)]. Lysates were incubated overnight at 4°C with 3 μg of rabbit antibodies against Runx2 antibody (M-70, Santa Cruz Biotechnology Inc.), and Suv39h1 (Santa Cruz Biotechnologies). Lysates were then incubated with protein A/G beads for 2 h, followed by four washes with wash buffer [50 mM NaCl, 20 mM Tris (pH 8.3), 0.5% Na-deoxycholate, 0.5% Nonidet P-40, 2 mM EDTA, 25 mM MG132, and 1× protease inhibitor mixture]. The total cell lysates and immunoprecipitated protein complexes were resolved by 8% SDS/PAGE and transferred to polyvinylidene difluoride membranes (Immobilon-P, Millipore, Billerica, MA). Blots were incubated with Runx2 (M-70) or Suv39h1 (C-14) antibodies. Membranes were then incubated with HRP-conjugated secondary antibodies against rabbit or mouse (1:2,000). Proteins bands were visualized with a chemiluminescence detection kit (Perkin–Elmer Life Sciences,Waltham, MA).

### Immunofluorescence

WI-38 and H1299 cells grown on gelatin coated cover slips were processed for immunofluorescence microscopy as previously described [31] using rabbit polyclonal Runx2 antibody (Santa Cruz Biotechnology, Inc.), followed by incubation with Alexa 488 conjugated secondary antibody (Molecular Probes, Eugene, OR). All images were taken using a Zeiss Axioplan digital microscope and analyzed using Metamorph software (Universal Imaging, Downingtown, PA).

## Abbreviations

BMP-3B, Bone morphogenetic protein-3B; DBD, DNA binding domain mutant; shRNA, Small hairpin RNA.

## Misc

Manish Tandon, Karthiga Gokul and Syed A Ali contributed equally to this work.

## **Competing interest**

The authors declare that they have no competing interests.

## **Author’s contributions**

MT and KG generated stable cell lines and analyzed cellular and molecular effects of Runx2 overexpression or knockdown (proliferation assay, wound healing assay, real time RT-PCR analysis, Western blots); SA performed ChIP and co-immunoprecipitation assays; ZC performed real time RT-PCR analysis and Western blots of samples with Runx2 overexpression or knockdown; JL and GS participated in the data analysis; JP conceived and coordinated the study and wrote the major part of the paper. All authors read and approved the final manuscript.
